# A Spiking Neural Model of HT3D for Corner Detection

**DOI:** 10.3389/fncom.2018.00037

**Published:** 2018-06-01

**Authors:** Pilar Bachiller-Burgos, Luis J. Manso, Pablo Bustos

**Affiliations:** Laboratory of Robotics and Artificial Vision, Department of Computer and Communication Technology, University of Extremadura, Cáceres, Spain

**Keywords:** spiking neural networks, hough transform, corner detection, feature detection, HT3D

## Abstract

Obtaining good quality image features is of remarkable importance for most computer vision tasks. It has been demonstrated that the first layers of the human visual cortex are devoted to feature detection. The need for these features has made line, segment, and corner detection one of the most studied topics in computer vision. HT3D is a recent variant of the Hough transform for the combined detection of corners and line segments in images. It uses a 3D parameter space that enables the detection of segments instead of whole lines. This space also encloses canonical configurations of image corners, transforming corner detection into a pattern search problem. Spiking neural networks (SNN) have previously been proposed for multiple image processing tasks, including corner and line detection using the Hough transform. Following these ideas, this paper presents and describes in detail a model to implement HT3D as a Spiking Neural Network for corner detection. The results obtained from a thorough testing of its implementation using real images evince the correctness of the Spiking Neural Network HT3D implementation. Such results are comparable to those obtained with the regular HT3D implementation, which are in turn superior to other corner detection algorithms.

## 1. Introduction

The Hough Transform (HT) (Hough, 1959) is a mathematical technique used as a means to detect lines and other features in computer images. The original algorithm consists of a procedure where pixels of interest (generally those corresponding to high frequencies) vote in a discretized parameter space to all the features it might correspond to (for instance, the line parameter space). The points of the parameter space with a number of votes above a threshold are considered actual features. Since its proposal, multiple variations of the original algorithm have been proposed, and the most widely-used line detection techniques rely on it.

The use of the HT for segment detection has been explored from different approaches over the last few decades. Two main approaches have been proposed. The first group of methods is based on image space verification from the information of the HT peaks (Gerig, 1987; Matas et al., 2000; Song and Lyu, 2005; Nguyen et al., 2008). The other group of methods deals with detecting properties of segments by analyzing the data in the parameter space (Cha et al., 2006; Du et al., 2010, 2012; Xu and Shin, 2014).

The Hough Transform has also been used for corner detection. Davies (1988) uses the Generalized Hough Transform (Ballard, 1987) to transform each edge pixel into a line segment. Corners are found in the peaks of the Hough space where lines intersect. Barrett and Petersen (2001) propose a method to identify line intersection points by finding collections of peaks in the Hough space through which a given sinusoid passes. Shen and Wang (2002) present a corner identification method based on the detection of straight lines passing through the origin in a local coordinate system. For detecting such lines, the authors propose a 1D Hough transform.

A variant using a three-dimensional parameter space named HT3D designed for the detection of corners, segments and polylines was presented in Bachiller-Burgos et al. (2017). The difference that makes HT3D outperform other state of the art algorithms is the voting process. Pixels vote for certain parts of lines instead of for whole lines. This allows this new transform to explicitly take into account segments and their endings. Although the HT has proven to be a relevant technique for the detection of multiple shapes and image features (Mukhopadhyay and Chaudhuri, 2014), it is worth noting that other methods exist for the joint detection of lines, contours, junctions, and corners. A remarkable alternative is the work presented in Buades et al. (2017) which, although biologically inspired, does not rely on neural networks. For a given image, their proposal computes the response of a pool of oriented filters and groups the result of the filters in a way that enables detecting contours, corners and T-junctions. Despite the aims of both methods being similar, in comparison to HT3D, the proposal of Buades et al. is not as versatile because it does not allow the direct detection of more complex shapes such as polygons.

H.B. Barlow postulated the use of the HT for feature detection in biological cortex in Barlow (1986), arguing that such a technique would allow to detect lines even with a limited capacity of neurons to establish connections with other neurons. G.G. Blasdel described a similar structure in the macaque monkey striate cortex in the context of orientation selectivity (Blasdel, 1992) and D. McLaughlin provided a detailed model using spiking neural networks (SNN) (McLaughlin et al., 2000). Okamoto et al. (1999) physiologically confirmed the existence of similar structures in macaque monkeys. Inspired by biological systems, different spiking neural models have also been proposed for different image processing tasks, including line detection using the Hough transform. Brückmann et al. (2004) presented a spiking neural network based on the HT for 2D slope and sinusoidal shape detection. After a training stage, the network is able to discriminate among different test patterns. A three-layered spiking neural network for corner detection was proposed in Kerr et al. (2011). The first layer corresponds to On/Off-center receptors. The neurons in the second layer take groups of adjacent neurons from the first layer as input and fire upon the detection of edges. The neurons of the third layer behave similarly with the neurons of the second layer and are supposed to be responsive to corners. The model presented in Weitzel et al. (1997) goes beyond biologically-plausible corner and line detection and proposes a spiking neural network which performs contour segmentation and foreground detection. It is based on grouping rather than any variation of the HT. An actual phenomenological interpretation for human vision was provided in Jacob et al. (2016). The proposed network learns to perform the HT and reproduces some optical illusions which also occur in humans. Others have also followed similar approaches to SNN performing the HT. That is the case of Wu et al. (2009), where a SNN for line detection is presented. A SNN for feature detection is also presented in Wu et al. (2007). In particular, the paper deals with *up-then-right* right angle corners. D.G. Lowe provides a biologically inspired model for object recognition in IT cortex where the Hough transform is used to generate object hypotheses (Lowe, 2000). A spiking neural network was applied to a Dynamic Vision Sensor (an event-based camera which only outputs changes in illumination) to detect and track lines using the HT in the work presented in Seifozzakerini et al. (2016).

In this paper, a spiking neural model of HT3D for corner detection is presented. The main motivation of our work is to extend the hypothesis of Blasdel about the existence of microcircuits performing the HT for orientation selectivity by introducing a biologically plausible neural model based on the HT for the detection of a variety of image features. The proposed neural network is mainly devoted to the detection of corners. Nevertheless, it provides the base topological neural structure on which new neural computations can give rise to the detection of more complex features. Likewise, the proposed SNN of HT3D provides an additional benefit in relation to the regular method from the point of view of a parallel execution. In this sense, the spiking implementation constitutes a parallel approach of the HT3D method that overcomes those aspects of the original algorithm limiting its parallelization.

The remainder of this article is organized as follows. Section 2 describes the HT3D transform. Its implementation as a spiking neural network is described in section 3. The experimental results are presented in section 4. To conclude, a discussion of the proposal and its performance is provided in section 5.

## 2. An overview of HT3D

**Figure 1:**
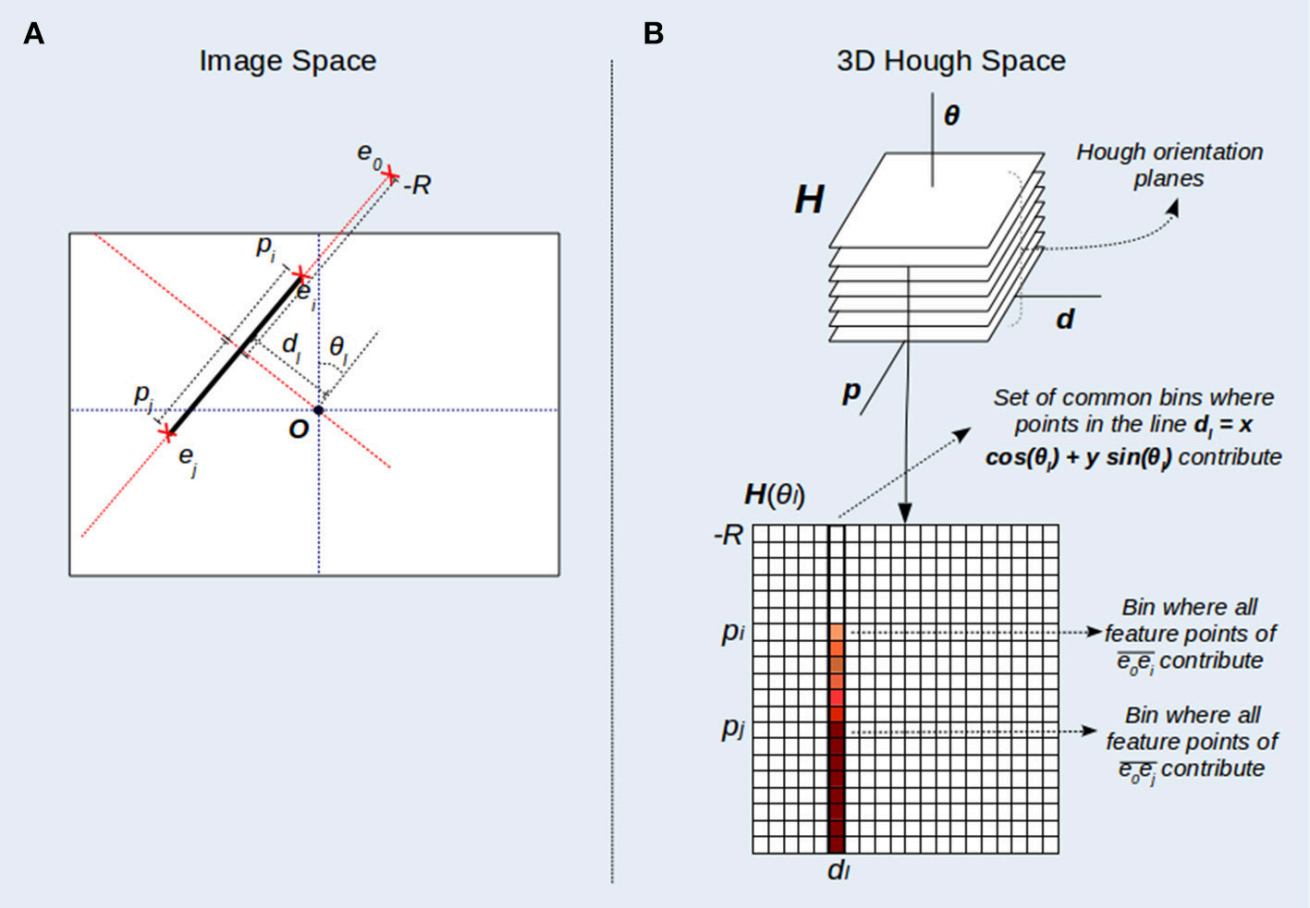
3D Hough space representation. **(A)** Pixel coordinates and values of the parameter *p* for points of a line *l*(*d*_*l*_, θ_*l*_). The value of *p* is computed by determining the *y-coordinate* of the point in a coordinate system local to the line *l* (dotted red lines). The image reference system (dotted blue lines) is situated at the image center (*O*). **(B)** Points of the line *l*(*d*_*l*_, θ_*l*_) contribute to a subset of cells situated at *d*_*l*_ in the Hough orientation plane *H*(θ_*l*_). Likewise, a point *e*_*i*_ situated at a position *p*_*i*_ relative to the line *l*(*d*_*l*_, θ_*l*_) contributes to the subset of cells for which *p*≥*p*_*i*_.

The Standard HT for straight line detection does not provide a direct representation of line segments, since feature points are mapped to infinite lines in the parameter space (Duda and Hart, 1972). To deal with segment representation, HT3D provides a 3D Hough space (Figure 1) that, unlike SHT, uses several cells to represent a line. This Hough space is parametrized by (θ, *d*, *p*), being θ and *d* the parameters of the line representation (*l*(*d*, θ) ≡ *d* = *x*cos(θ) + *y*sin(θ)) as in the standard HT. The additional parameter *p* defines positions of the possible segment endpoints relative to each line. It is assumed that the origin of the image coordinate system is located at its center. Thus, θ ∈ [0, π), and *d, p* ∈ [−*R*, +*R*], with *R* being the half of the length of the image diagonal. To compute the relative position *p* of each point of a given line, a coordinate system local to the line is considered, where the vertical axis coincides with the line and the horizontal one passes through the image origin (see Figure 1A). Using this local system, the relative position (*p*) of a point *e* = (*x, y*) of the line *l*(*d*, θ) can be computed by determining the *y-coordinate* of the point as follows:

(1) $$\begin{array}{r} p=-x\sin\left(\theta \right)+y\cos\left(\theta \right) \end{array}$$

In this new parameter space (see Figure 1B), a cell (θ, *d*, *p*) represents an image segment of the line *l*(*d*, θ), defined by the point pair *e*_0_ − *e*, with *e*_0_ being a fixed endpoint situated at the smallest line position (*p*_0_ = −*R*) and *e* a variable endpoint situated at any position *p* within the line *l*. Since *p* > = *p*_0_, an image feature point *e*_*i*_ = (*x, y*) belongs to the segment $\bar{e_{0}e}$ if it is a point of the line *l* (Equation 2) and its relative position in the coordinates of the line (its corresponding *p*_*i*_ parameter) is lower or equal than *p* (see expression 3). Thus, any point (*x, y*) contributes to those cells (θ, *d*, *p*) in the 3D Hough space that verifies:

(2) $$\begin{array}{r} d=x\cos\left(\theta \right)+y\sin\left(\theta \right) \end{array}$$

and

(3) $$\begin{array}{r} p>=-x\sin\left(\theta \right)+y\cos\left(\theta \right) \end{array}$$

According to this, in order to perform the HT3D transform, each feature point must vote for those cells verifying expressions 2 and 3 for every orientation plane (*H*(θ)). Since this voting process is computationally expensive, it is divided into two steps:

- In the first step, feature points only vote for the first segment they could belong in each orientation plane (i.e., *p* is computed using only the equality of expression 3)
- Once the first vote of each feature point for every orientation plane has been performed, starting from the second lower discrete value of *p*, each cell in *H*(θ_*d*_, *d*_*d*_, *p*_*d*_) accumulates with *H*(θ_*d*_, *d*_*d*_, *p*_*d*_−1), being θ_*d*_, *d*_*d*_ and *p*_*d*_ discrete values of θ, *d* and *p*, respectively.

This reduces the computational cost of this phase of the algorithm, producing the same result as the complete voting scheme in which each image point votes for all the cells fulfilling expressions (2) and (3).

### 2.1. Feature representation in the 3D hough space

The 3D Hough space provides a compact representation of any image feature that can be defined in terms of line segments. This representation emerges from the implicit relation between image line segments and Hough cells. Thus, for any pair of pixels, there is an associated pair of Hough cells that allows the estimation of the number of points included in the corresponding image segment. Specifically, given two points *e*_*i*_ = (*x*_*i*_, *y*_*i*_) and *e*_*j*_ = (*x*_*j*_, *y*_*j*_) of a line *l*(*d*_*l*_, θ_*l*_) and being *p*_*i*_ and *p*_*j*_ the relative positions of *e*_*i*_ and *e*_*j*_ within the line *l* according to Equation (1), the number of feature points included between *e*_*i*_ and *e*_*j*_ can be computed as:

(4) $$\begin{array}{r} H_{i↔j}=|H\left(\theta_{l},d_{l},p_{i}\right)-H\left(\theta_{l},d_{l},p_{j}\right)| \end{array}$$

with *H* being the 3D Hough space.

This measure can be used to determine the likelihood of the existence of an actual image segment between both points. In a similar way, for more complex image features, such as polygons, the combination of information provided by different orientation planes gives rise to a quantification of the degree of presence of that feature in each image region. This has been applied to rectangle detection in Bachiller-Burgos et al. (2017).

Besides the representation of line segments and polylines, the 3D Hough space provides a distinctive representation of characteristic image points such as corners, since they can be treated as segment intersections that produce specific configurations of cells in the Hough space. Thus, in general, any segment endpoint, including corners, can be detected from local cell patterns of the Hough space, as described in the next section.

### 2.2. Detection of segment endpoints with HT3D

**Figure 2:**
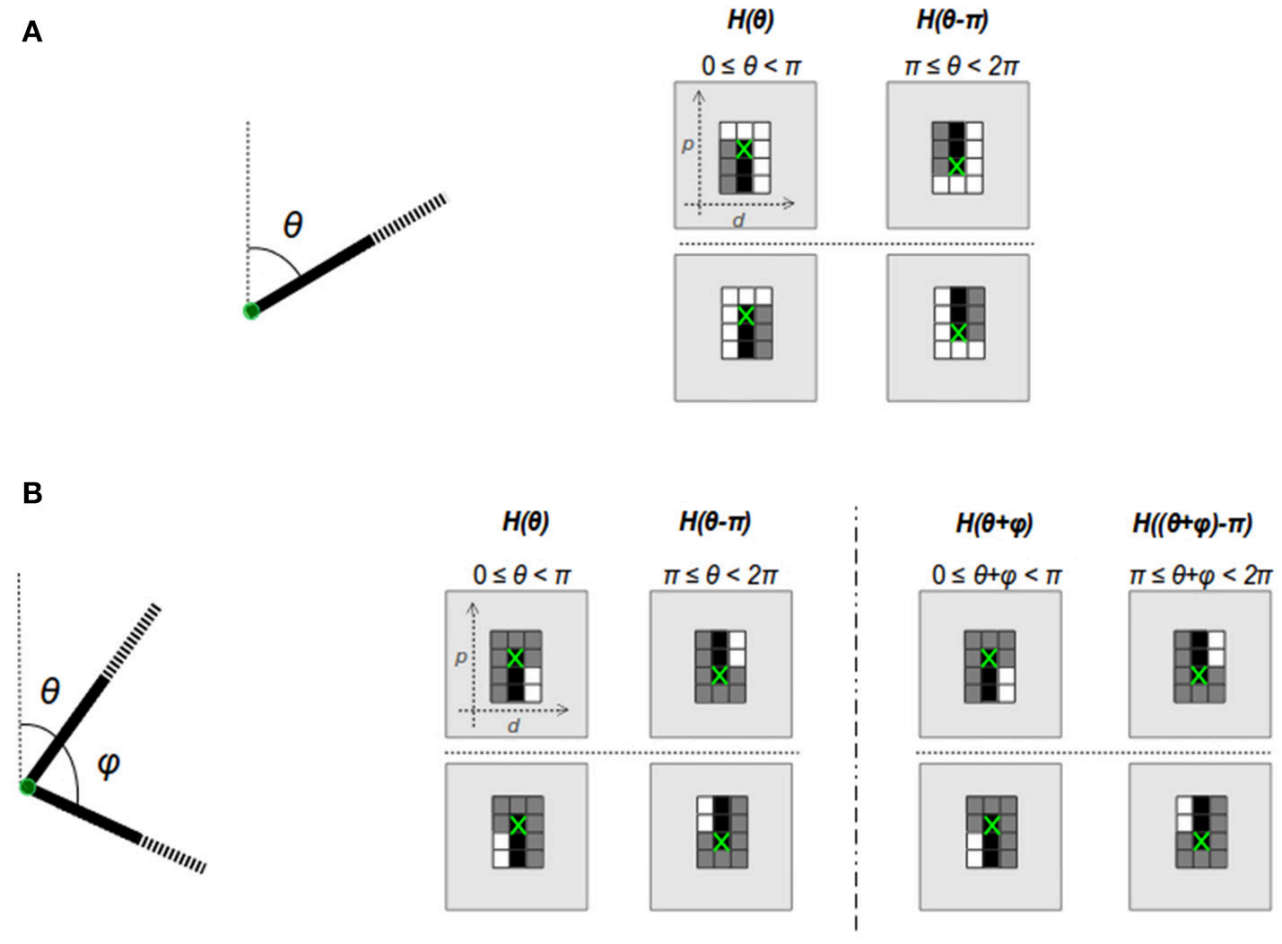
Cell patterns of the 3D Hough space for non-intersection endpoints **(A)** and corners **(B)**.

Segment endpoints are classified into two groups in HT3D: corners and non-intersection endpoints. Corners are line segment intersections. Non-intersection segment endpoints correspond to endpoints which do not meet other segments. Considering the image line segments defining both kind of points, specific cell patterns can be observed in the 3D Hough space. These cell patterns are depicted in Figure 2.

Image line segments characterizing a corner or endpoint transform into vertical segments of cells in the corresponding orientation planes of the 3D Hough space, since the value of the parameter *d* remains constant for all the points of the same image line. This fact is represented by the black cells of Figure 2, which correspond to cells whose values differ from the ones above them. To limit the size of the cell patterns, a fixed length is considered for the piece of segments taking part in corner and endpoint detection, since it is not necessary to check complete segments for determining the existence of both kind of points. Beside black cells, corner and endpoint patterns include white cells representing empty line sections^1^, i.e., cells with similar content, and gray cells that correspond to cells whose content can be ignored. For each kind of pattern, a flipped version is also considered in order to cover the orientation range [π, 2π).

To check patterns of corner and non-intersection endpoints, cells in the 3D Hough space are grouped into vertical full (black cells) or empty (white cells) segments. A vertical full segment verifies that the difference between its last and first cell is greater than a threshold τ_*F*_. On the other hand, this difference must be lower than a near to zero threshold (τ_*E*_) in an empty segment. Thus, given a certain position in the discrete Hough space (θ_*d*_, *d*_*d*_, *p*_*d*_) and taking η as the number of cells of a full piece of segment in the Hough representation, to verify if that position contains, for instance, a corner, the following must be fulfilled:

(5) $$\begin{array}{r} H\left(\theta_{d},d_{d},p_{d}\right)-H\left(\theta_{d},d_{d},p_{d}-\eta \right)>\tau_{F} \end{array}$$

and

(6) $$\begin{array}{r} H\left(\theta_{d},d_{d}+1,p_{d}-1\right)-H\left(\theta_{d},d_{d}+1,p_{d}-\eta \right)<\tau_{E} \end{array}$$

or

(7) $$\begin{array}{r} H\left(\theta_{d},d_{d}-1,p_{d}-1\right)-H\left(\theta_{d},d_{d}-1,p_{d}-\eta \right)<\tau_{E} \end{array}$$

for the plane *H*(θ_*d*_) and the same expressions for the corresponding position of the plane *H*((θ+φ)_*d*_), given a certain range of φ and assuming θ < π and (θ+φ) < π.

Once Hough cells containing corners and non-intersection endpoints are detected, the intensity image is used to find the most likely pixel position associated with the corresponding position in the Hough space. To this end, the set of image positions corresponding to the Hough cell *H*(θ_*d*_, *d*_*d*_, *p*_*d*_) is approximated using an image window centered on (*x*_*c*_, *y*_*c*_):

(8) $$\begin{array}{r} x_{c}=d\cos\left(\theta \right)-p\sin\left(\theta \right) \end{array}$$

(9) $$\begin{array}{r} y_{c}=d\sin\left(\theta \right)+p\cos\left(\theta \right) \end{array}$$

with θ, *d*, and *p* being the real values associated with θ_*d*_, *d*_*d*_, and *p*_*d*_. The window size is defined by the resolutions of *p* and *d* used to create the discrete Hough space. Considering this window, the pixel position of a corner or endpoint is located by searching for that pixel maximizing some corner/endpoint criterion. Specifically, the minimum eigenvalue of the covariance matrix of gradients over the pixel neighborhood is used. This coarse-to-fine approach avoids applying any threshold related to changes of intensity in the point local environment, which allows the identification of feature points that cannot be detected by other methods.

## 3. The proposed spiking neural model for corner detection

This section details the proposed spiking neural model for corner detection based on HT3D. Besides corners, the proposal includes the detection of non-intersection segment endpoints because some corners may present a great correspondence to the Hough patterns of this kind of points. This is the case of corners with low intensity in one of their edges and corners with low acute angles.

**Figure 3:**
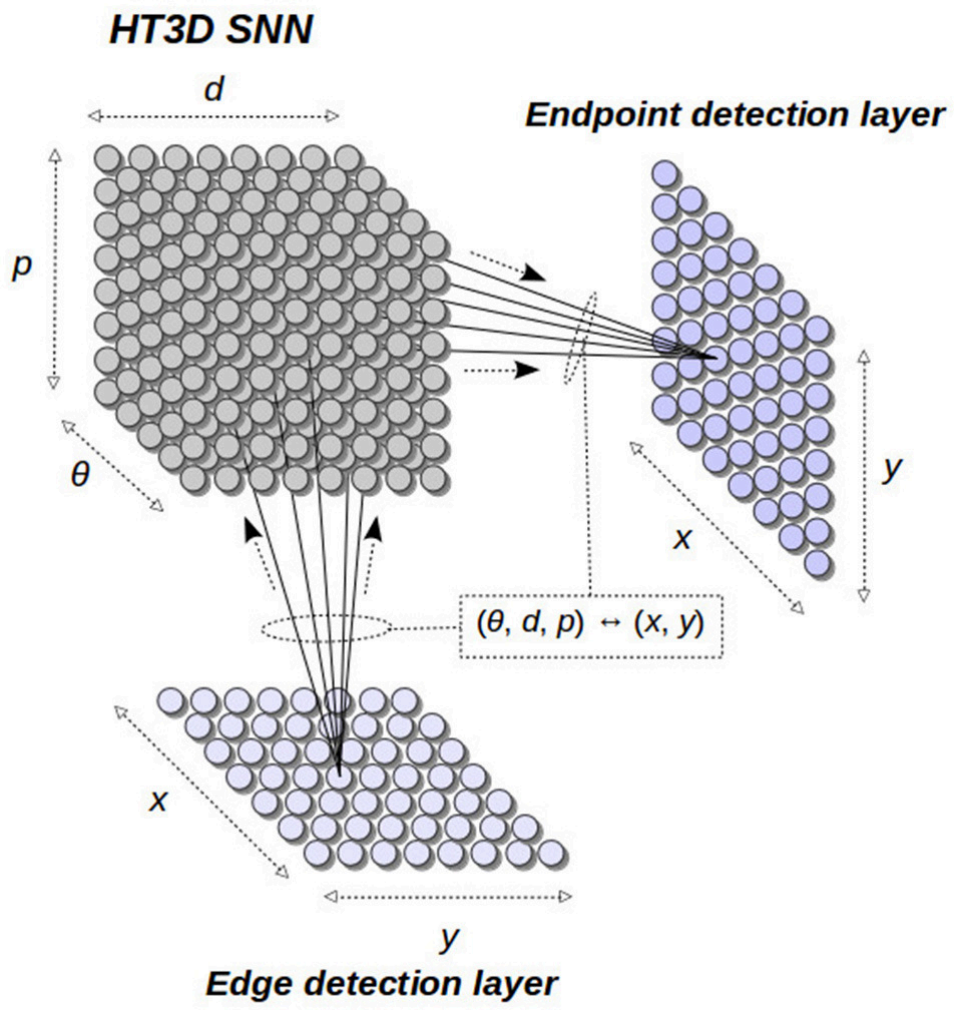
General structure of the proposed spiking neural model. The 3D Hough space is implemented as a 3D SNN connected to an input edge detection layer and an output endpoint detection layer. Connections between the HT3D SNN and the other two layers are established according to the relation between Hough cell positions and image positions.

The general structure of our neural model is depicted in Figure 3. The 3D Hough space is represented by a 3D network of spiking neurons, receiving its input from an edge detection layer and sending its output to an endpoint detection layer. The neurons in these two layers are directly related to the image points in the *x* − *y* plane. Thus, each neuron of these layers represents an image position. On the other hand, neurons of the HT3D SNN represent discrete positions of the 3D Hough space of the form (θ, *d, p*). This way, connections between this SNN and the two *x* − *y* arrays are established according to the relation between (θ, *d, p*) and (*x, y*).

Spiking neurons in our proposal are implemented using the Leaky Integrate and Fire (LIF) neuron model (Burkitt, 2006). Thus, given a spiking neuron *n* with *N* inputs ($n_{j}^{\left(I\right)}$), being each input a spike train, and an output (*n*^(*O*)^), *n* produces a spike whenever its membrane potential *P*_*n*_ reaches a certain threshold Θ_*n*_. According to the LIF model, at time *t*, the membrane potential *P*_*n*_(*t*) is updated as follows:

(10) $$\begin{array}{r} P_{n}\left(t\right)=P_{n}^{\prime }\left(t\right)+\overset{N}{\underset{j=1}{\sum }}w_{jn}n_{j}^{\left(I\right)} \end{array}$$

with *w*_*jn*_ the synaptic weight of the *j*th input and being $P_{n}^{\prime }\left(t\right)$ defined by:

(11) $$\begin{array}{r} P_{n}^{\prime }\left(t\right)=sign\left(P_{n}\left(t-\Delta t_{s}\right)\right)\max\left(|P_{n}\left(t-\Delta t_{s}\right)|-\lambda \Delta t_{s},0\right) \end{array}$$

The last equation models the “leak” of the membrane potential, being Δ*t*_*s*_ the time interval between the last input spike and the current one and λ the rate of linear decay (Seifozzakerini et al., 2016).

From a computational point of view, the following aspects are also considered in the spiking neuron model employed:

- The membrane potential is updated only when an input spike is received (Lee et al., 2016).
- Once a spike is generated in the output of the neuron, the membrane potential is reset to zero (resting potential). A zero refractory period is applied for simplicity.
- Despite synaptic weights of biological neurons are non-negative (Maas, 1997) , both positive and negative weights are considered in our neuron model with the aim of reducing the number of required neurons^2^.

The proposed neural architecture is composed of these basic neural units. The values of synaptic weights and firing thresholds are established according to the specific function of the neurons in the network.

### 3.1. The HT3D spiking neural network

The main structure of the HT3D spiking neural network consists of a series of decoupled layers of spiking neurons (Hough orientation layers), acting as Hough orientation planes of the 3D parameter space. Thus, for a given orientation layer, all its neurons represent a discrete value of θ. In addition, orientation layers as a whole represent the complete range of angles between 0 and π.

**Figure 4:**
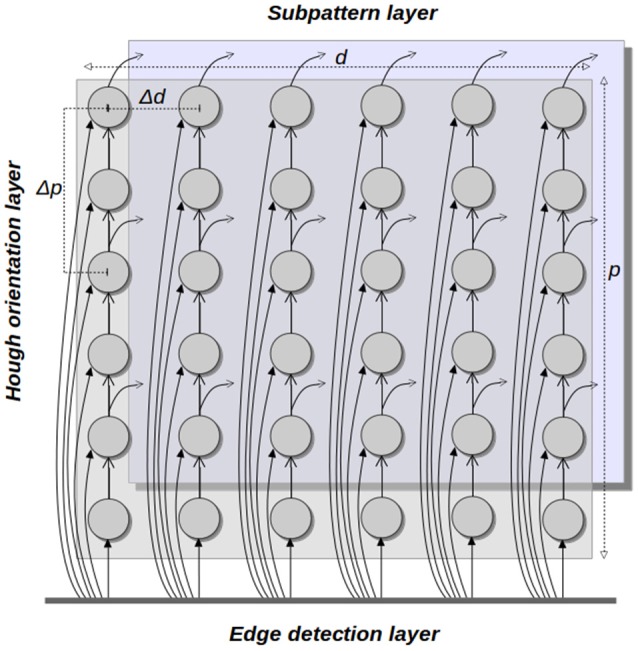
Hough orientation layer of the HT3D SNN.

Neurons of these orientation layers, called Hough neurons, represent cells of the 3D Hough space. Within each layer, neurons are disposed in different columns (see Figure 4), being each column an image line representation in the parameter space. Thus, neurons of the same column share a common discrete value of the parameter *d*. Likewise, adjacent columns represents a separation of Δ*d* in the value of *d*, being Δ*d* the quantization step of such a parameter. In a given column, neurons correspond to different values of the parameter *p* of the associated line.

The output of each Hough neuron *h*_θ*dp*_ must provide a spiking encoding of the votes associated to the corresponding cell (θ, *d, p*), according to expressions 2 and 3. This entails defining a temporal relation between votes of a Hough cell and spikes generated by its neural counterpart. To this end, neurons are firstly excited at once by the outputs of the edge detection layer through the synapses established by the following equations:

(12) $$\begin{array}{r} d=x\cos\left(\theta \right)+y\sin\left(\theta \right) \end{array}$$

(13) $$\begin{array}{r} p=-x\sin\left(\theta \right)+y\cos\left(\theta \right) \end{array}$$

These synapses correspond to the contribution of features points to the first segment they could belong to in a given line (equality of expression 3). As in the original algorithm, this only covers the first stage of the voting process. To deal with the contribution of feature points to the remaining Hough cells defined by the greater operator in expression 3, each neuron sends its output to the next neuron of the same column through a feedforward synapse. This synapse provides a propagation of the signals received by a given neuron along the subsequent neurons of the same column, which equates to the accumulation stage of the voting process of the original HT3D algorithm. Through spike propagation, votes of a Hough cell are processed by the corresponding neuron in different time steps. Thus, each neuron generates a spike train that represents the votes of the associated Hough cell.

In order to maintain a near 1 to 1 correspondence between cell votes and spikes generated by the corresponding neuron, each neuron of a column represents an increase of 1 pixel in the value of *p* with respect to the previous neuron. Nevertheless, a quantization step for *p* (Δ*p*) greater than 1 pixel can be used for representing the discrete Hough space. In such case, only the outputs of certain rows are used by the neural units in charge of processing the information of Hough neurons, while the function of the neurons of the remaining rows is limited to spike propagation (see Figure 4).

### 3.2. Neural processing of pieces of segment of corner and endpoint patterns

As in the original HT3D, corner and endpoint pattern detection requires checking every full or empty piece of segment of the different kind of patterns by separately analyzing the corresponding pairs of cells of the 3D Hough space. This is accomplished in the proposed SNN by a new type of spiking neurons, called subpattern neurons.

**Figure 5:**
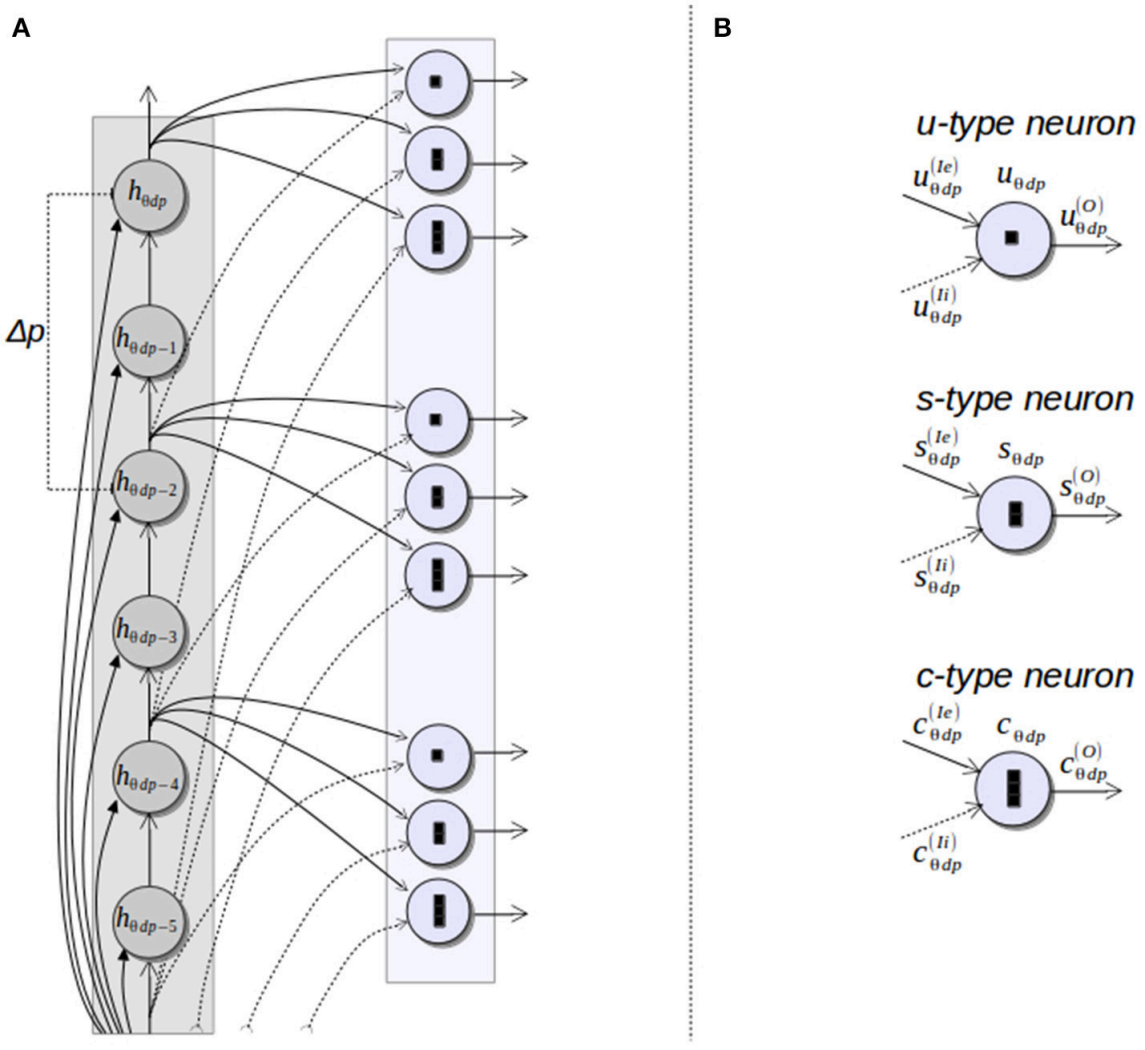
Subpattern neurons. **(A)** Connections between a column of a Hough orientation layer and the corresponding subpattern neurons; **(B)** types of subpattern neurons.

In order to provide the necessary information about the pieces of segment composing corner and endpoint patterns, the neurons that represent discrete positions of the Hough space, according to a quantization step of Δ*p* for the parameter *p*, send their outputs to subpattern layers. Specifically, neurons of each column of a Hough orientation layer located with a separation of Δ*p* in the row position are connected to a subpattern layer representing the same orientation. Subpattern layers are in charge of processing significant pieces of the patterns of corners and non-intersection endpoints. With this aim, three types of subpattern neurons are considered in these layers: u–type, s–type and c–type neurons (Figure 5). Neurons of these three types “count” the number of votes of a piece of segment of a certain length from a given position of the 3D Hough space. Thus, u-type neurons consider a length of Δ*p* and correspond to cells on the top and at the bottom of an endpoint pattern in its normal and flipped form, respectively (see Figure 2A). Likewise, s-type neurons are related to the empty pieces of segment at both sides of the corner patterns (Figure 2B). The neurons of this type consider a segment length of (η − 1)Δ*p*, with η the number of cells of a full piece of segment in both, corner and endpoint patterns. Lastly, c-type neurons accumulate votes for pieces of segment with a length of ηΔ*p* providing information about empty pieces of segment at both side of the endpoint patterns, as well as about full pieces of segment at the central column of every pattern.

**Figure 6:**
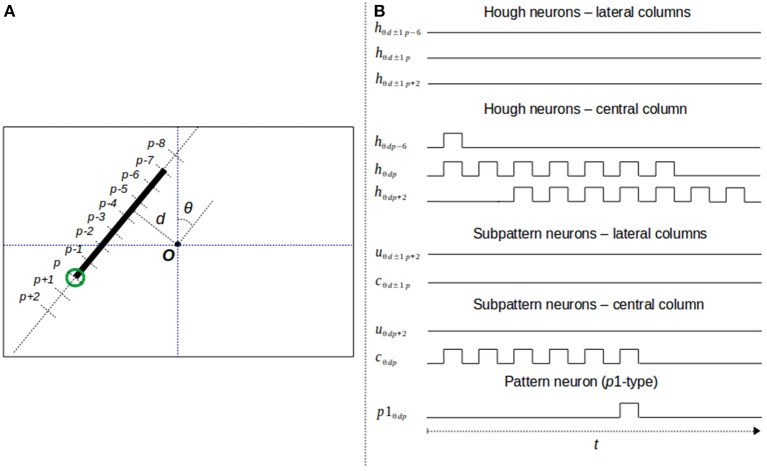
Neural response to a segment endpoint. **(A)** Segment endpoint in the image space; **(B)** output of the neurons taking part in the detection process.

The three types of subpattern neurons produce spike trains that represent the difference of votes between the Hough cells *H*(θ, *d, p*) and *H*(θ, *d, p* − *l*), being *l* the length of the piece of segment considered by the specific type of neuron. To this end, each neuron has an excitatory input and an inhibitory one. The excitatory input is connected to the output of the Hough neuron *h*_θ*dp*_, representing the Hough cell *H*(θ, *d, p*). The inhibitory input is connected to the output of the neuron *h*_θ*dp*−*l*_ through a synapse with a negative weight of the same magnitude than the one of the excitatory synapse. To synchronize the spike trains of both inputs, the inhibitory synapse introduce a delay in the signal related to the value of *l*. For instance, assume that two spikes are simultaneously generated by the neurons *h*_θ*dp*_ and *h*_θ*dp*−Δ*p*_ and travel through the synapses that connect these two neurons to the u-type neuron *u*_θ*dp*_. If the spike from *h*_θ*dp*_ influences the neuron *u*_θ*dp*_ at time *t*, the effect of the spike from *h*_θ*dp*−Δ*p*_ on the neuron *u*_θ*dp*_ occurs at time *t*+*t*_*ms*_Δ*p*, being *t*_*ms*_ the minimum time interval between two consecutive spikes. This synaptic delay, along with the complementary excitatory and inhibitory connections, cancels the spikes representing common votes of the Hough cells *H*(θ, *d, p*) and *H*(θ, *d, p* − Δ*p*). As a consequence, *u*_θ*dp*_ generates a spike train that can be interpreted as the votes received by the piece of segment of the line *l*(θ, *d*) defined between the relative positions *p* and *p* − Δ*p*. Figure 6 shows an example of this behavior for several u-type and c-type neurons, considering Δ*p* = 2 and η = 3. A complete description of this figure is provided in the next section.

### 3.3. Neural detection of corners and non-intersection endpoints in the 3D hough space

**Figure 7:**
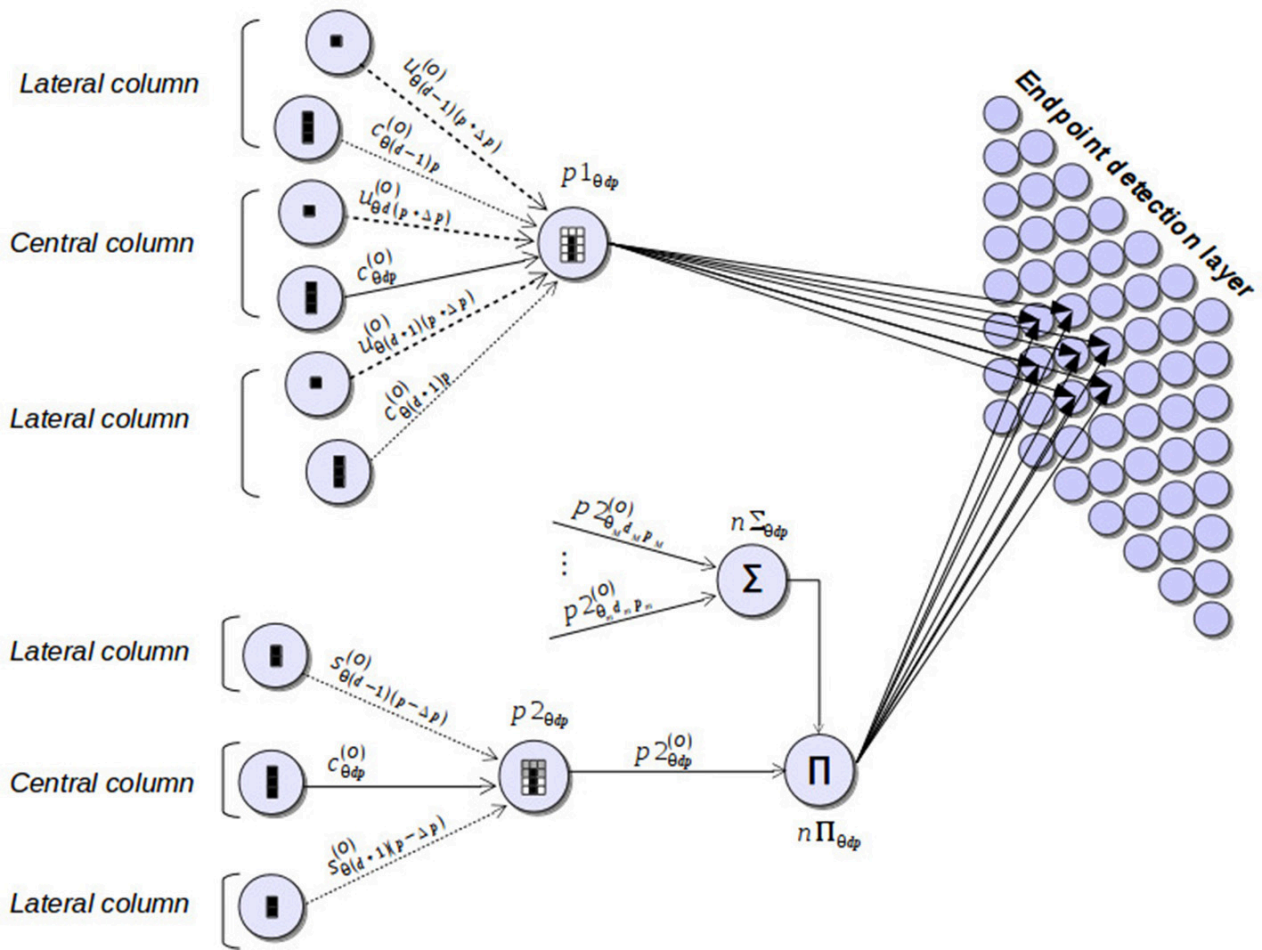
Neural components taking part in the final stage of the corner and endpoint detection process. Pattern neurons (*p*1 and *p*2) combine the outputs of subpattern neurons, producing a spike whenever a corner or endpoint pattern is detected. These neural responses excite the endpoint detection layer by means of synapses that provide a mapping between Hough positions and image positions.

Complete patterns of corners and non-intersection endpoints are detected by another group of neurons (pattern neurons) that combine the outputs of subpattern neurons. Each pattern neuron generates a spike whenever the pieces of segment that have been processed by the corresponding subpattern neurons fit the associated pattern. Two types of pattern neurons are considered: *p*1 neurons, in charge of verifying the patterns of non-intersection endpoints, and *p*2 neurons, which check corner patterns of one of the two orientation planes defining the point. Figure 7 illustrates these two types of neurons and the subnet connections that give rise to the final stage of the detection process. In this figure, sign and magnitude of synaptic weights are symbolized by arrows with different style and thickness. Specifically, negative weights are represented by dotted-line arrows, while continuous-line arrows refer to positive weights. In addition, line thickness is an indicator of the magnitude of the weight. Thus, the greater the thickness of the line, the greater the magnitude of the weight.

Pattern detection is accomplished by *p*1 and *p*2 neurons, for each position of the discrete Hough space, considering the information of the different pieces of segment defining the corresponding pattern. This information is obtained from the outputs of subpattern neurons and is organized in three columns, two lateral columns and a central one, as is depicted in Figure 2. The central column corresponds to the column of the orientation layer where the pattern is checked. The lateral columns are the two adjacent columns to the central one.

For the detection of an endpoint pattern, a *p*1 neuron considers two different pieces of segment for each column: the one defined by the top cell of the column (bottom cell in the flipped version of the pattern) and the one defined by the remaining η cells of the column. As can be observed in Figure 2A, for the three columns, the top cells in the normal version of the pattern or the bottom cells in the flipped one should represent empty pieces of segment. To check this condition, pattern neurons of type *p*1 are connected to the u-type neurons representing the three top/bottom cells through highly inhibitory weights (see Figure 7). This implies that the three cells have to represent empty pieces of segment to fit the pattern.

The information about the pieces of segment defined by the remaining η cells of the three columns of an endpoint pattern is obtained from the outputs of the corresponding c-type neurons. As is shown in Figure 2A, lateral cells should not represent full pieces of segment at both sides of an endpoint pattern. With the aim of identifying this situation, lateral c-type neurons are connected to *p*1 through inhibitory synapses, but in this case, the magnitude of the weights is much smaller than the one of the connections from u-type neurons. This means that the detection of lateral pieces of segment has a penalization effect over the detection of an endpoint pattern, but this penalization does not prevent from detecting the pattern. It just suppresses neural responses to points corresponding more likely to edges rather than segment endpoints. Regarding the η cells of the central column, they have to represent a full piece of segment to fit an endpoint pattern. To verify this condition, a *p*1 neuron is connected to the c-type neuron associated to the central column through an excitatory synapse. Weights of the inhibitory connections are established on the basis of the weight of this excitatory synapse according to the following equations:

(14) $$\begin{array}{r} w_{uI}=-\eta \Delta pw_{cE} \end{array}$$

(15) $$\begin{array}{r} w_{cI}=-\rho_{p1}w_{cE} \end{array}$$

with *w*_*cE*_ being the weight of the excitatory synapse, *w*_*uI*_ the weight of the connection from a u-type neuron, *w*_*cI*_ the weight of the inhibitory synapses from a lateral c-type neuron and ρ_*p*1_ the penalization factor associated with lateral pieces of segments. The firing threshold of a *p*1 neuron (Θ_*p*1_) is related to the length of the piece of segment of the central column taking also into account the leak term of the neuron, the tolerance to segment gaps and the allowed penalization for points in adjacent segments. Assuming *w*_*cE*_ = 1, Θ_*p*1_ can be set to ηΔ*p* − ξ_*p*1_, with ξ_*p*1_ being a value quantizing the aforementioned causes of reduction of the membrane potential.

Neurons of type *p*2, devoted to checking corner patterns in a given orientation plane, take their inputs from s-type and c-type neurons (Figure 7). Through these connections, a *p*2 neuron processes the pieces of segment of the three columns of the pattern (Figure 2B) applying the same principles used in *p*1 neurons for the detection of endpoint patterns. Specifically, connections from s-type neurons provide information about the pieces of segment of the lateral columns of the corner pattern. These connections are inhibitory, penalizing the pattern detection in a similar way as lateral c-type neurons do in endpoint detection. The excitatory synapse from a c-type neuron to the corresponding *p*2 neuron facilitates the identification of the full piece of segment of the central column of a corner pattern. Excitatory (*w*_*cE*_) and inhibitory (*w*_*sI*_) presynaptic weights of a *p*2 neuron maintain a similar relation to the one of Equation (15):

(16) $$\begin{array}{r} w_{sI}=-\rho_{p2}w_{cE} \end{array}$$

Equation (16) represents the corresponding penalization introduced by non-empty pieces of segments at both sides of a corner pattern, taking ρ_*p*2_ as the penalization factor. Similarly to *p*1 neurons, for *w*_*cE*_ = 1, the firing threshold Θ_*p*2_ can be established as ηΔ*p* − ξ_*p*2_, with ξ_*p*2_ being a quantization of the tolerance to line gaps and non-empty adjacent segments, considering the leak term of the neuron as well.

Complete detection of corners is accomplished by combining the outputs of the *p*2 neurons of the different orientation layers according to the range of angles defining a corner point. Specifically, the response of a *p*2 neuron of the orientation layer that represents an angle θ is multiplied (neuron *n*_Π_) by the linked response (neuron *n*_Σ_) of the *p*2 neurons representing the same image position in the 3D Hough space for the range of angles [θ_*m*_, θ_*M*_], with (θ_*m*_−θ) and (θ_*M*_−θ) the minimum and maximum angles of a corner, respectively. Thus, an active neural response representing the detection of a corner in a certain position will only take place in the presence of at least two firing neurons of *p*2 type, one of them associated with an angle θ and another one representing an angle in the range [θ_*m*_, θ_*M*_].

To illustrate the whole neural process taking part in the detection of a segment endpoint, Figure 6 shows an example of the neural behavior of the HT3D SNN considering the endpoint marked in green in Figure 6A. The image space representation of Figure 6A also shows the relation between image and Hough positions of the concerned line segment. Thus, as can be observed, the position in the 3D Hough space of the marked endpoint that is relevant for its detection correspond to (θ, *d, p*). This means that the endpoint detection produces a neural response in the neuron *p*1_θ*dp*_.

In this example, values of 2 and 3 are considered for Δ*p* and η, respectively. Therefore, the pattern neuron *p*1_θ*dp*_ receives its inputs from the subpattern neurons *u*_θ*d*−1*p*+2_, *u*_θ*dp*+2_, *u*_θ*d*+1*p*+2_, dealing with the top cells of the endpoint pattern, and *c*_θ*d*−1*p*_, *c*_θ*dp*_ and *c*_θ*d*+1*p*_, in charge of processing the lateral and central pieces of segment of the pattern. Likewise, each u-type neuron is connected to two neurons of a column of the corresponding Hough orientation layer. These two neurons represent a separation of 2 (Δ*p*) of the parameter *p*. Thus, for instance, the neuron *u*_θ*dp*+2_ is connected to the neuron *h*_θ*dp*+2_ through an excitatory synapse and to the neuron *h*_θ*dp*_ through an inhibitory one. Similarly, c-type neurons are fed by two Hough neurons that represent a separation of 6 (ηΔ*p*) within the corresponding column of the 3D Hough space. This way, the neuron *c*_θ*dp*_ is connected to *h*_θ*dp*_ and *h*_θ*dp*−6_.

Figure 6B shows the output of all the aforementioned neurons considering the line segment of Figure 6A. The output of each Hough neuron represents the votes of the corresponding cell of the 3D Hough space. The neurons of the lateral columns produce no spike since there are no feature points in the associated lines of the image. On the other hand, neurons of the central column, the one associated to the line segment of the endpoint, generate spike trains that correspond to the accumulation of votes of each Hough cell. Regarding subpattern neurons, they produce spike trains representing the number of points of a piece of segment from the associated Hough position. u-type neurons consider pieces of segment of length 2 and c-type units segments with a length of 6 pixels. As can be observed, since the input of the inhibitory synapse of these neurons is delayed according to the length of the associated piece of segment, neuron *u*_θ*dp*+2_ remains inactive. The absence of a neural response indicates that the top cell of the central column is empty. On the contrary, the neural processing of neuron *c*_θ*dp*_ generates a spike train formed by 6 spikes, which represents the existence of a full piece of segment in the central column. All the subtpattern neurons connected to *p*1_θ*dp*_, excepting *c*_θ*dp*_, are inactive during the time window required for pattern detection. Since the only excitatory connection of *p*1_θ*dp*_ is the one that links the pattern neuron to *c*_θ*dp*_, the membrane potential of the pattern neuron increases with every spike generated by the subpattern neuron. Considering a firing threshold of 4 and taking into account the membrane potential decay, the neuron *p*1_θ*dp*_ fires once the fifth spike from *c*_θ*dp*_ is received. The generated spike constitutes the neural response to the detection of a segment endpoint in the Hough position (θ, *d, p*).

### 3.4. Neural detection of corners and non-intersection endpoints in the image space

The detection of corners and non-intersection endpoints at this stage of the neural process results in the identification of the positions of the 3D Hough space that match the corresponding patterns. In order to produce neural responses that identify detected features in the image space, neural detectors of segment endpoints of the HT3D SNN send their outputs to the endpoint detection layer.

The neurons of the endpoint detection layer represent pixel positions and are laterally connected to provide surround inhibition. Connections between the HT3D SNN and this layer are established according to the coordinate transformation expressed in Equations (1) and (2), taking into account the discretization of the parameter space. Thus, a detector neuron of the HT3D SNN representing a Hough position (θ, *d, p*) sends its output to those neurons of the endpoint detection layer (*e*_*xy*_) that represent pixel coordinates (*x, y*) for which the corresponding discrete position in the Hough space for an angle θ, $\left(\theta_{d},d_{d}^{\prime },p_{d}^{\prime }\right)$, coincides with (θ_*d*_, *d*_*d*_, *p*_*d*_), the discrete counterpart of (θ, *d, p*). Through these connections, a neuron *e*_*xy*_ increases its membrane potential according to the evidence of the existence of a segment endpoint in its near surrounding area. Thus, neurons that exhibit the greatest membrane potential in their local neighborhoods represent the most likely pixel positions of corners and non-intersection endpoints.

Responses from neurons of the endpoint detection layer representing non-maximum locations of segment endpoints are suppressed using a local lateral inhibition approach. Thus, each neuron is connected to the neighboring neurons located within a window of size *w* × *w*. By means of these connections, whenever a neuron fires, its surrounding neurons are reset, suppressing any possible active response from them. To improve the accuracy of the detection results and reduce false positives, neurons taking part in this non-maximum suppression process can be limited to those neurons representing edge pixel positions. This can be achieved by connecting each neuron of the edge detection layer to the corresponding neuron of the endpoint detection layer. These connections allow “enabling” only certain neurons of the endpoint detection layer and ensure that every detected point corresponds to an edge pixel.

This strategy for locating segment endpoints in the image space is similar to the one presented by Barrett and Petersen (2001). Despite this approach differs from the final stage of the original HT3D algorithm, the idea of a reverse voting from the Hough space to the image space adapts in a direct and simple way to SNN and produces similar results to the ones obtained by the regular implementation of HT3D.

### 3.5. Computational requirements of the proposed spiking neural network

In order to provide an analysis of the computational requirements of the proposed spiking neural network, three different measures are considered: number of neurons, number of input connections of each neuron and firing latency period, measured as the time interval that a neuron takes to process the sequences of incoming spikes until it finally fires. The different types of neurons are organized in layers representing orientation steps. For each type of layer, the computational requirements are specified below, taking *n* and *m* as the number of discrete steps of the parameters *d* and *p*, respectively, and Δ*t*_*p*_ as the minimum time interval taken by a neuron to process all its inputs. It is assumed that Δ*t*_*p*_ does not depend on the number of inputs of a neuron, since that number is one of the measures of this analysis.

- **Hough orientation layers**: the number of neurons of each layer of this type is *n* × *m* × Δ*p*, since, for each column, discrete steps of 1 pixel are considered for the parameter *p*. Each neuron has *n*_*e*_ + 1 inputs, with *n*_*e*_ being the number of connections from the edge detection layer according to the coordinate transform between image and Hough spaces. The additional input corresponds to the feedforward connection that provides the propagation of spikes (votes) through the neurons of each column. Regarding the firing latency, neurons of these layers can fire with a period of Δ*t*_*p*_, since their basic function is to propagate every incoming spike through their corresponding columns.
- **Subpattern layers**: layers of this type consist of *n* × *m* × 3 neurons, as three subpatterns have to be processed for each position of the discrete Hough space. Each neuron has two inputs, one excitatory and one inhibitory, and a firing latency period of Δ*t*_*p*_.
- **Patterns layers**: for each position of the discrete Hough space, patterns of corners and endpoints have to be checked in both normal and flipped versions. In addition, corner detection requires 2 more neurons (*n*_Σ_ and *n*_Π_). Thus, the number of neurons of each pattern layer is *n* × *m* × 8. Neurons of type *p*1 and *p*2 have, respectively, 6 and 3 inputs and require a processing time of η * Δ*t*_*p*_, being this time interval an upper bound. The two additional neurons used to confirm complete corner patterns, *n*_Σ_ and *n*_Π_, process *l*_*c*_ and 2 inputs, respectively, with *l*_*c*_ being the number of orientation planes defining the rank of angles of corners. Both types of neurons fire with a latency period of Δ*t*_*p*_.

The total number of layers of each type is given by the angular resolution Δθ. Following the original HT3D algorithm, the maximum angular resolution is defined by the next equation:

(17) $$\begin{array}{r} \Delta \theta =\arctan\frac{1}{\eta \Delta p} \end{array}$$

This provides an inverse relation between the number of neurons of each layer and the total number of layers. Thus, the increase in the number of neurons of each layer produced by low values of Δ*p* is compensated with a reduction of the number of layers.

Besides this 3-dimensional neural structure, the endpoint detection layer is composed of *w*×*h* neurons, with *w* and *h* being the width and height of the image. Considering only the connections from the HT3D SNN, the neurons of this layer receive four inputs from each pattern layer, representing the neural responses in the presence of corners and non-intersection endpoints. Assuming that all these neural responses are simultaneously received, the firing latency of these neurons is Δ*t*_*p*_.

As can be observed, in general, the different types of neurons of the HT3D SNN have a reduced number of inputs, which indicates that the computational cost of the neural processing of each individual neuron is certainly low. Nevertheless, the total number of required neurons is clearly high, producing a global computational cost that exceeds the one of many other existing methods for corner detection. A sequential programming approach could consider the existence of many inactive neurons or even entire columns of the Hough orientation layers associated to image lines with few or no edge pixels. This would reduce the processing times of a sequential implementation, however the number of operations would still be superior to those of the original algorithm, since the regular HT3D can process a corner/endpoint subpattern using a single subtraction operation while the firing latency of pattern neurons is proportional to η.

The main advantage of the spiking implementation emerges when considering a parallel implementation using specific hardware (Schuman et al., 2017). Thus, even using a parallel implementation, certain phases of the original HT3D algorithm must be sequentially performed, such as the accumulation stage of the voting process. On the contrary, a parallel spiking implementation allows the overlap of the different phases of the algorithm. This way, the propagation of spikes/votes through the columns of the Hough orientation layers, which is the equivalent neural process to the accumulation of votes, can be carried out at the same time as the detection of corner/endpoint patterns. Considering an extension of the proposed neural model for the detection of more image features such as line segments and rectangles, the phase overlapping provided by the spiking execution would increase even more the difference in performance between the original and the spiking implementation of HT3D, since all the different image features could be detected in the time window needed for spike propagation.

## 4. Results

A simulated model of the proposed spiking neural network has been tested using the *YorkUrbanDB* dataset (Denis et al., 2008). This dataset is formed by 102 marked images of urban environments. Each image has an associated set of ground truth line segments corresponding to the subset of segments satisfying the *Manhattan assumption* (Coughlan and Yuille, 2003). To evaluate our proposal, detected corners and endpoints of the proposed neural model have been compared with the segment endpoints of the ground truth data. In order to show how our approach can outperform intensity-based detection methods, results from the Harris corner detector (Harris and Stephens, 1988) have also been obtained, comparing the set of detected points with the ground truth data.

**Figure 8:**
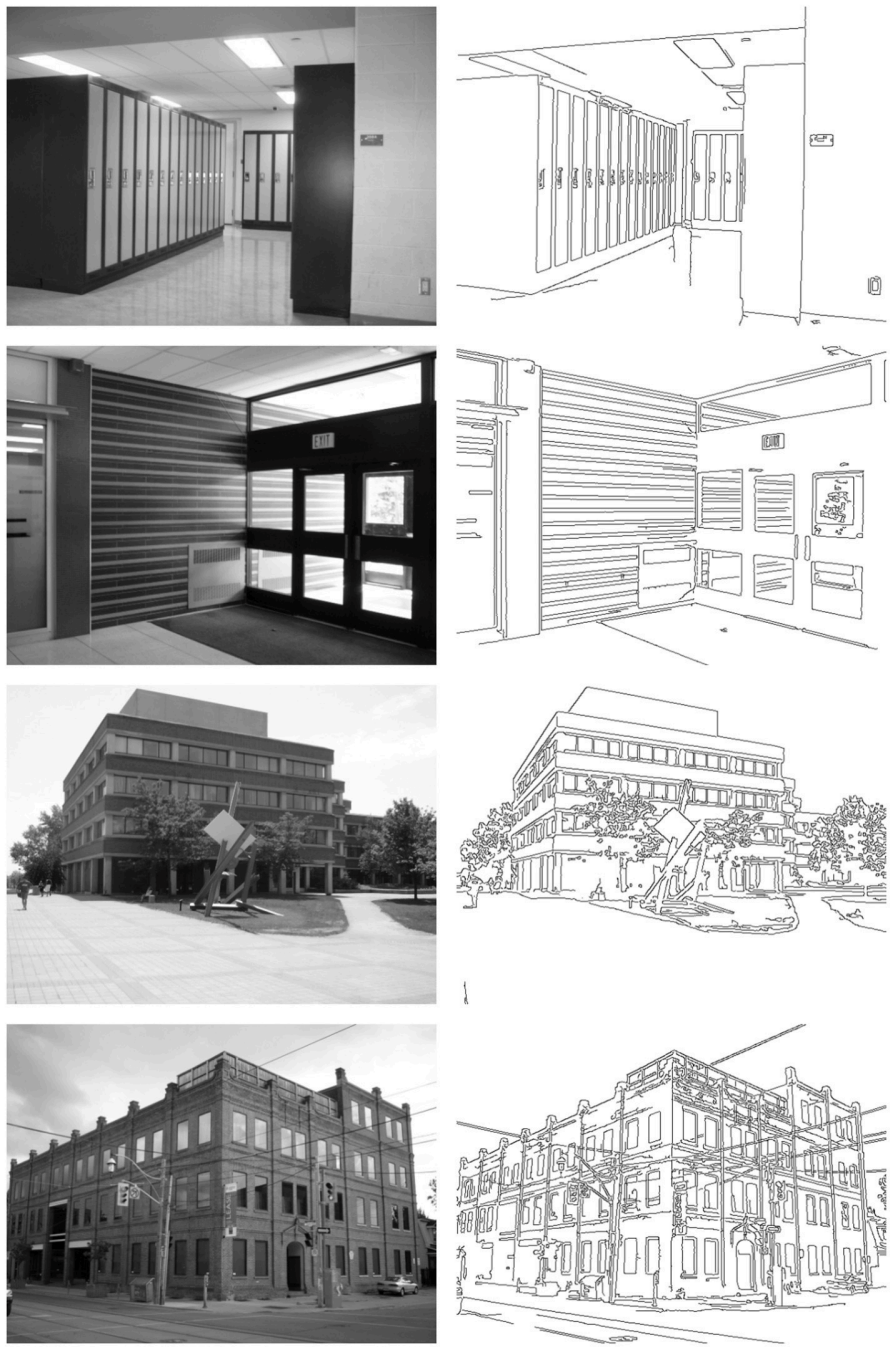
First representative subset of images used to evaluate the proposed neural model. Original images are shown in the **left** column. **Right** column depicts the corresponding edge images.

**Figure 9:**
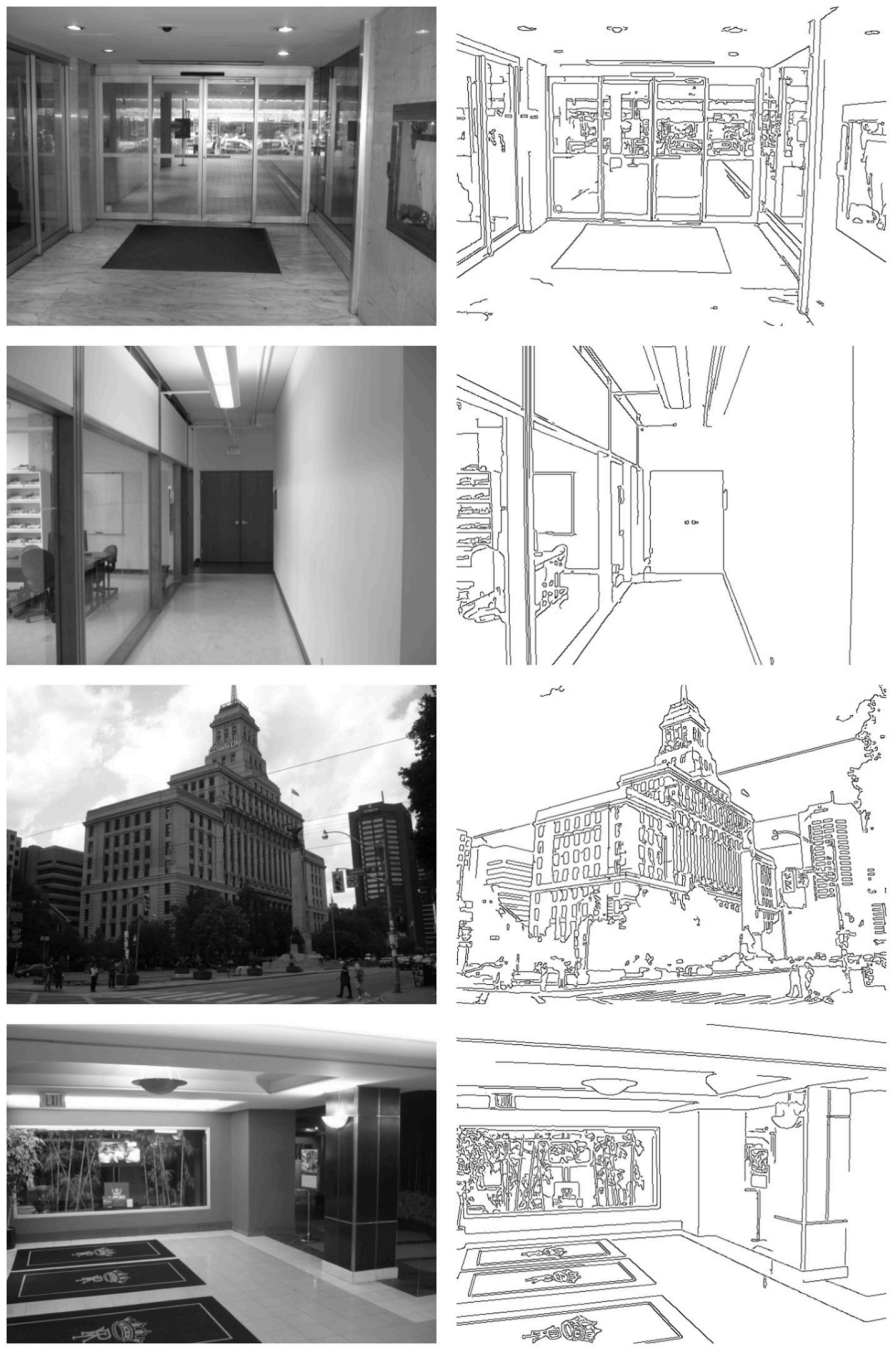
Second representative subset of images used to evaluate the proposed neural model. **Left** column shows the original images. The corresponding edge images are shown in the **right** column.

In this section, a quantitative evaluation of our method obtained using the whole *YorkUrbanDB* dataset is presented. In addition, detailed detection results for a representative subset of images are shown. This subset corresponds to the eight images of Figures 8, 9. These figures also show an image representation of the edge detection layer feeding the HT3D SNN for each test.

**Figure 10:**
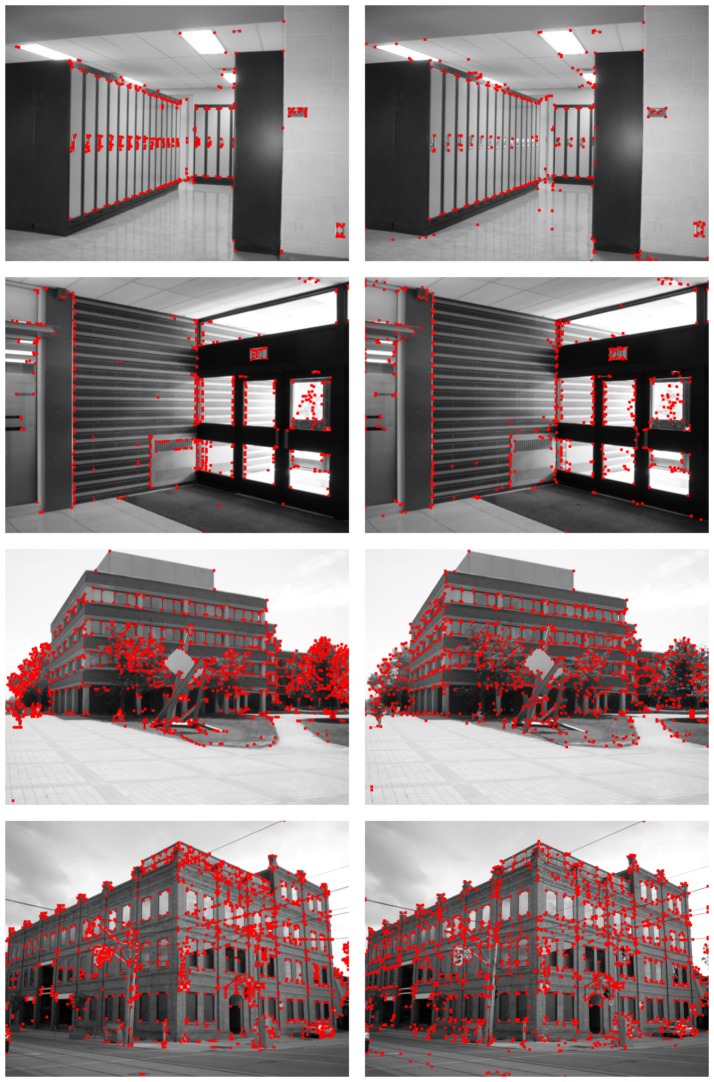
Detection results of the first subset of images using the Harris corner detector (**left** column) and the proposed SNN (**right** column).

**Figure 11:**
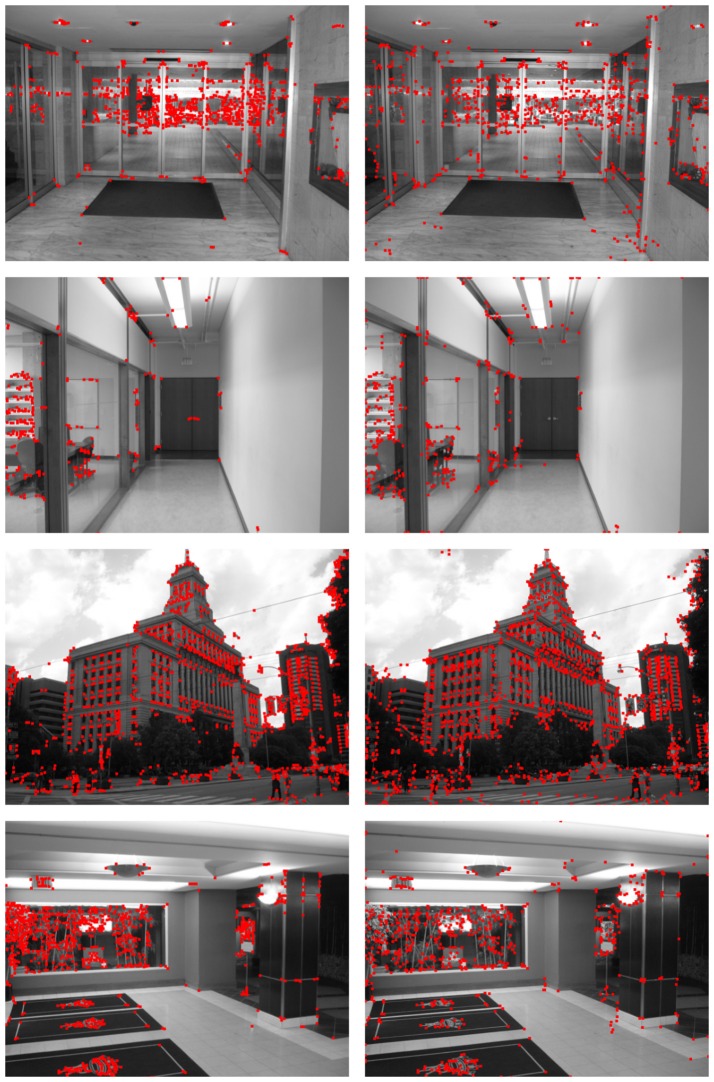
Detection results of the second subset of images using the Harris corner detector (**left** column) and the proposed SNN (**right** column).

Figures 10, 11 show the detection results for the eight test images using Harris (left column) and the proposed SNN (right column). For the Harris detector, a window size of 5 pixels and a sensitive factor of 0.04 have been chosen. For the proposed HT3D SNN, a value of 2 has been fixed for Δ*d* and Δ*p*^3^ and a total of 79 orientation steps (Δθ = 0.04) have been considered. Also, the number of cells of full pieces of segments in the patterns of corners and non-intersection endpoints (η) has been set to 6 and an angle range between 35 and 145° has been used for corner detection. According to the original algorithm, the angular resolution for corner and endpoint detection can be established using (Equation 17), which, for the chosen values of η and Δ*p*, produces an approximate angular resolution of 0.08. Nevertheless, we use half of this angle for Δθ in order to ensure each feature is detected in more than one orientation layer. This way, neurons of the endpoint detection layer representing actual positions of corners and non-intersection endpoints are excited by more neurons of the HT3D SNN than those of the surrounding area of true corners/endpoints.

Regarding synaptic weights and neuron parameters, results presented here have been obtained considering the following:

- The rate of decay of the membrane potential (λ) has been set to 0.2/*t*_*ms*_, with *t*_*ms*_ being the minimum time interval between two consecutive spikes.
- Presynaptic weights of *p*1 pattern neurons have been fixed according to expressions 14 and 15, considering a value of 1 for *w*_*cE*_ and a penalization factor ρ_*p*1_ of 0.25. Thus, the inhibitory weights *w*_*uI*_ and *w*_*cI*_ have been set to −12 and −0.25, respectively.
- Presynaptic weights of pattern neurons of *p*2 type have been established, as in *p*1 neurons, using (Equation 16) for *w*_*cE*_ = 1 and ρ_*p*2_ = 0.25.
- The firing thresholds of the two types of pattern neurons have been set to 6.5 for Θ_*p*1_ and 8 for Θ_*p*2_. The difference between both thresholds is motivated by the fact that endpoint patterns detected by neurons of *p*1 type include more adjacent cells than corner patterns.

From Figures 10, 11, the differences between the detection results of the Harris detector and the proposed HT3D-based neural model can be appreciated. While right angle corners are correctly detected by both methods, only the proposed model performs well in the detection of obtuse angle corners. In addition, as expected, only our approach detects non-intersection endpoints. These points are mainly found in the intersections between image segments and image limits, but they also correspond to corners with low intensity in one direction.

**Figure 12:**
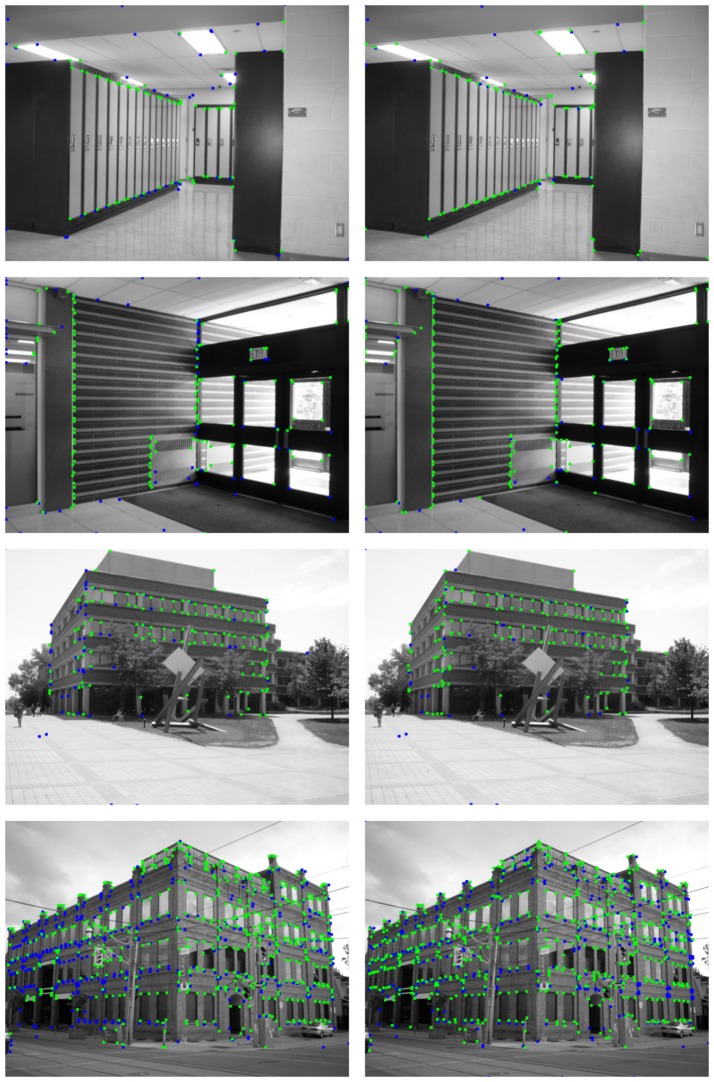
Matching between ground truth endpoints of images in Figure 8 and detected corners/endpoints using the Harris corner detector (**left** column) and the proposed SNN (**right** column). Green squares represent correct matchings. Ground truth endpoints with no correspondence with detected points are drawn as blue squares.

**Figure 13:**
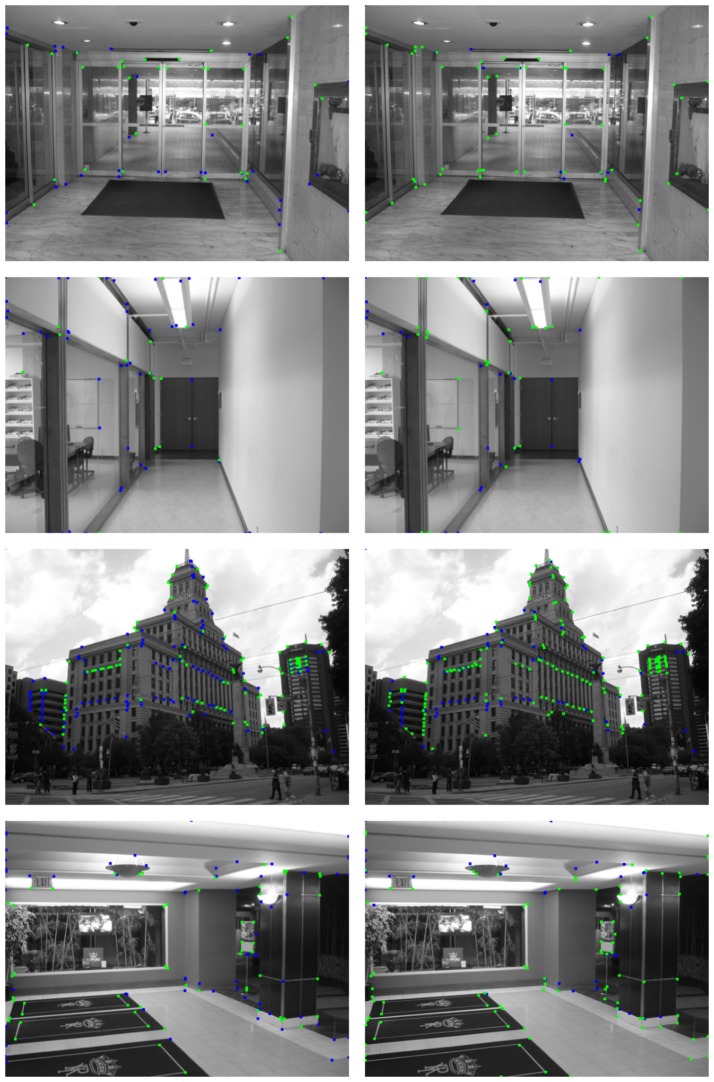
Matching between ground truth endpoints of images in Figure 9 and detected corners/endpoints using the Harris corner detector (**left** column) and the proposed SNN (**right** column). Correct matchings are represented as in Figure 12.

The differences between both methods are more noticeable when the detection results are compared to the ground truth data, as can be observed in Figures 12, 13. These figures show the segment endpoints of the ground truth data and their correspondences with the detections of the Harris method (left column) and the proposed neural model (right column). To obtain the correct matchings between detected and ground truth endpoints, a maximum euclidean distance of 3 pixels between each pair of points has been considered. Distances have been rounded to integer values in order to approximate the acceptance area to a circular image patch. The resulting correct matchings are shown as green squares. Blue squares represent ground truth endpoints with no correspondence with any detected endpoint according to the distance criterion. As can be seen in these figures, the hit rates of the HT3D-based model are around 15−25% over the ones obtained by Harris in all the test images. This difference is mainly related to the aforementioned additional points detected by our approach, although some difference in accuracy can also be observed. Thus, in some cases, despite a Harris corner is detected in a near position to a ground truth endpoint, the distance between them is above the allowed distance, which denotes a greater accuracy of our proposal in comparison to the Harris method.

**Figure 14:**
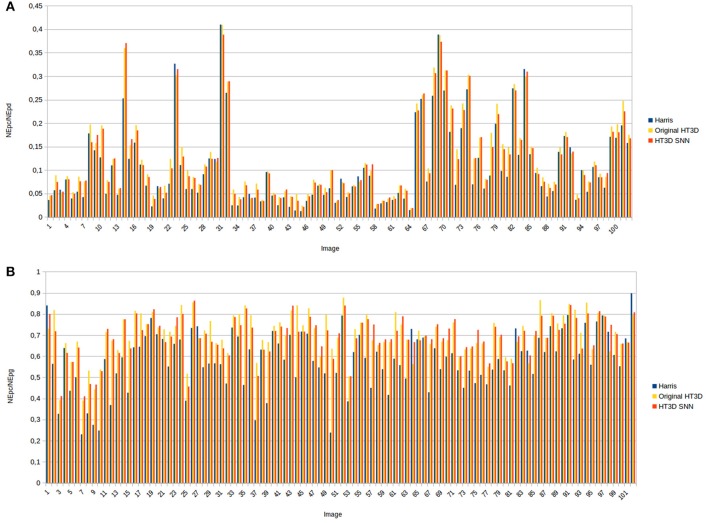
Quantitative results obtained from the whole *YorkUrbanDB* dataset for the Harris detector, the original HT3D algorithm and the proposed SNN. **(A)** Ratio between the number of correct matchings with the ground truth (*NEp*_*c*_) and the total number of detections (*NEp*_*d*_); **(B)** ratio between the number of correct matchings and the total number of ground truth points (*NEp*_*g*_).

The above observations can be extended to the great majority of images of the dataset as can be appreciated in Figure 14. This figure shows, for each image, the ratio between the number of correct matchings with the ground truth (*NEp*_*c*_) and the total number of detected points (*NEp*_*d*_) and the hit rate, i.e., the ratio between *NEp*_*c*_ and the number of ground truth endpoints (*NEp*_*g*_). A maximum rounded distance of 3 pixels between detected and ground truth points has been established to compute *NEp*_*c*_. The two ratios have been obtained for the proposed spiking neural model, the Harris corner detector and the original HT3D algorithm^4^. The ratio *NEp*_*c*_/*NEp*_*d*_ produces moderate values for the three methods since the ground truth endpoints correspond to a subset of corners and segment endpoints and all the methods detect additional points. Nevertheless, this measure acts as a validity indicator of the hit rate. Thus, a low value of this ratio for one detector in relation to other methods suggest a high number of false positives that invalidate a high hit rate. In general, the three methods produce comparable ratios between the correct matchings and the total number of detections, although larger values can be observed for HT3D in both the spiking implementation and the original one (Figure 14A). This is mainly due to the fact that the number of correct matchings with the ground truth is noticeably superior in the HT3D approach as indicated by the hit rate (Figure 14B). Regarding the two implementations of HT3D, only small variations of the two ratios are appreciated between the two methods. Thus, the accuracy of both approaches can be considered similar, proving the correctness of the proposed neural model.

Performance of an HT-based approach is strongly tied to the discretization of the parameter space. Thus, choosing suitable values for Δ*d*, Δ*p*, and Δθ is crucial to obtain reliable results. The decision is not as simple as reducing these resolutions as much as possible, since fine quantizations increase noise sensitivity. In order to reach an equilibrium between accuracy of the results and robustness against image noise, parameter resolutions must be established according to the nature of the image. In HT3D, the distribution of edge points determines the proper values of Δ*d* and Δ*p*. Thus, close parallel line segments can only be correctly represented in the 3D Hough space using low values of these quantization steps. However, if edge point chains are more sparsely distributed in the image, larger values produce comparable detection results.

This fact can be observed in Table 1. This table shows the values of *NEp*_*d*_ (number of detected corners/endpoints) and *NEp*_*c*_ (number of correct matchings) for the 8 test images using the HT3D SNN with different quantization steps. The total number of ground truth endpoints is shown in the first column as a reference. As can be appreciated, increasing Δ*d* and Δ*p* affects the hit rates of images 4 and 7 to a greater extent, since they are the most complex images in terms of edge distribution, while the hit rates of the remaining images do not suffer so significant variations. Indeed, the number of correct matchings of images 5 and 6 remains almost constant.

**Table 1:** Detection results of the HT3D SNN for the 8 test images using different values of Δ*d*, Δ*p*, and Δθ.

		**Δ*****d*** = 2 **Δ*****p*** = 2 **Δθ** = 0.04		**Δ*****d*** = 3 **Δ*****p*** = 3 **Δθ** = 0.04		**Δ*****d*** = 2 **Δ*****p*** = 2 **Δθ** = 0.08	
	***NEP*_*g*_**	***NEP*_*d*_**	***NEp*_*c*_**	***NEP*_*d*_**	***NEp*_*c*_**	***NEP*_*c*_**	***NEp*_*d*_**
1	111	232	86	189	79	211	83
2	199	495	156	467	146	458	153
3	190	1,179	152	838	134	983	146
4	959	1,572	611	1,101	497	1,373	575
5	69	842	57	634	57	731	56
6	63	370	37	324	36	325	35
7	219	1,596	149	1,068	89	1,296	131
8	127	732	92	532	83	561	84

*For each configuration, the table shows the total number of detected corners/endpoints (NEp_d_) and the number of correct matchings with the ground truth (NEp_c_). The number of ground truth points (NEP_g_) is shown in the first column*.

Regarding the angular resolution Δθ, a proper value for corner/endpoint detection can be established by means of Equation (17), once Δ*p* and η have been fixed. This provides an upper bound for this quantization step, although smaller values could favor the non-maximum suppression neural process of the endpoint detection layer as previously stated. Table 1 illustrates this point. The last two columns of this table show the number of detected points and correct matchings using the maximum angular resolution for Δ*p* = 2 and η = 6 (0.08 radians). As it can be observed, in general, the hit rates diminish with regards to the results obtained using an angular step of 0.04. Nevertheless, this reduction is much less noticeable in comparison with the one produced when increasing the other two quantization steps. In addition, the number of operations considerably decreases with respect to the other two configurations of Table 1, producing an additional benefit in terms of computational requirements. For images with a moderate density of edge points, further reductions of the size of the Hough space can lead to similar results. Thus, for images 5 and 6, fixing a value of 3 for Δ*d* and values of 2 and 0.08 for Δ*p* and Δθ, respectively, produces equivalent hit rates to the ones obtained with the first configuration, using one third of the neurons required by such configuration.

## 5. Discussion

SNN have gained an increasing interest in fields such as image processing and computer vision. These models are more biologically plausible and have proven to be more powerful than those of the previous generations of neural networks. Biological findings about the existence of cortical structures performing similar computations to that of the Hough Transform have inspired various spiking neural models for line and corner detection.

Based on a novel variant of the Hough Transform (HT3D) that provides a combined detection of corners, line segments and polylines, we have presented in this paper a new spiking neural network for corner detection that can be naturally extended for the detection of additional image features. HT3D employs a 3D Hough space that offers a compact representation of line segments. This parameter space also encloses canonical configurations of segment endpoints, distinguishing between two kinds of points: corners and non-intersection endpoints. Through these cell configurations, the detection of these feature points can be solved by means of a pattern matching strategy.

The proposed spiking neural model consists of a 3D spiking neural network (HT3D SNN) connected to two layers of neurons representing image positions (the edge detection layer and the endpoint detection layer). The edge detection layer feeds the HT3D SNN with the outputs of an edge detection neural network. The endpoint detection layer responds to the identification of Hough patterns of segment endpoints in the image space. Neurons of the HT3D SNN represents discrete positions of the Hough space. Thus, connections between the HT3D SNN and the other two layers of neurons of the neural model are established according to the relation between Hough cell positions and image positions. In the proposed HT3D SNN, the 3D Hough space is implemented as decoupled layers of spiking neurons associated to possible discrete values of a line orientation. Each column of these layers corresponds to an image line representation in the Hough space. Spikes generated by a given neuron are propagated to the subsequent neurons of the same column. Through this propagation, each neuron generates a spike train that encodes the votes of the corresponding cell of the Hough space. Pairs of these spike trains are fed to another group of neurons called subpattern neurons. These neuronal units are in charge of processing significant pieces of segment of corner and endpoint patterns. Specifically, they produce spike trains representing the difference in votes of two Hough cells, i.e., the number of votes of a given piece of segment, using complementary inhibitory and excitatory presynaptic weights along with synaptic delays. Outputs of subpattern neurons are connected to other neurons responsible of detecting corner and non-intersection endpoint patterns. Spikes generated by this set of neurons excite the endpoint detection layer through synapses that provide a mapping between Hough positions and image positions. Neurons of this final layer are responsive to corners and non-intersection endpoints in the image space and are laterally connected in order to suppress responses from neurons representing non-maximum locations of segment endpoints.

The proposed neural model has been tested by means of a software simulation using a set of real images labeled with ground truth line segments. A comparison with the Harris corner detector has also been conducted to show the benefits of our approach in relation to intensity-based corner detection methods. Results obtained are comparable to the ones provided from the original algorithm which demonstrates the correctness of our neural model. Comparison with the ground truth evinces superior hit rates of our proposal with regard to the Harris detector, which are mainly related to non-intersection endpoints and obtuse angle corners, but also denote greater accuracy of our neural approach. We have also shown the influence of the size of the Hough space in the detection results. According to the experiments, for non-complex images, a significant reduction on the number of neurons can lead to comparable hit rates.

As shown in the experiments, the spiking implementation of HT3D does not provide additional benefits in terms of accuracy in relation to the original algorithm. Nevertheless, a hardware implementation of the proposed SNN can improve the time performance of a parallel execution of the regular HT3D method. Thus, sequential stages of the algorithm can overlap with other stages in the spiking implementation. This overlapping allows a better exploitation of the parallelism, which leads to lower processing times.

From both theoretical and experimental perspectives, we have demonstrated that the principles that associate the neural processes taking place in orientation selectivity to cellular microcircuits implementing a Hough-like transform can be extended to create neural structures responsive to other kind of features. The proposed spiking neural network constitutes a base neural structure that maps visual stimuli sets onto neural representations of a variety of line-based features. Although our model is mainly focused on corner detection, it provides the basic neural organization to address the identification of other image features. Thus, as in the regular HT3D method, line segment information is encoded in pairs of neurons (Hough cells) of each column of a Hough orientation layer. According to this, a similar approach to the one employed by subpattern and pattern neurons to process and detect pieces of segment can be used to extend the proposed model to the detection of more complex features such as line segments and polygons. Thus, for a given pair of image points *e*_*i*_ and *e*_*j*_ of a line *l*(θ, *d*), neural responses to a line segment between those points can be obtained by means of a new neural unit taking its inputs from the associated pair of neurons of the corresponding column of the HT3D SNN representing the line *l*. Combining synaptic delays, complementary excitatory and inhibitory synapses and an appropriate relation between the length of the line segment and the firing threshold of the neuron, neural units exhibiting a suitable behavior for segment detection can be implemented. This constitutes a direct extension of our model, although it should be complemented with an appropriate neural representation of the additional detected image features. In this sense, a more complex neural model than the one used in the current approach should be applied to provide a generalized neural representation of image features. An interesting line of exploration is related to the theories supporting the temporal correlation of neural activity as a mean of dealing with perceptual grouping and sensory segmentation (von der Malsburg and Buhmann, 1992; von der Malsburg, 1994; Singer and Gray, 1995). Our future work points to this direction with the main objective of providing a complete neural implementation of HT3D.

## Author contributions

All authors contributed ideas to the design and implementation of the proposal. PB-B and LM wrote the first draft of the manuscript. PB modified the content of the manuscript. All authors read and approved the final version of the manuscript.

### Conflict of interest statement

The authors declare that the research was conducted in the absence of any commercial or financial relationships that could be construed as a potential conflict of interest.

## Footnotes

^1^White cells situated beside black cells allow reducing the number of false corners and endpoints caused by nearby image segments.

^2^Signed weights can be substituted by pairs of excitatory and inhibitory neurons (Maas, 1997).

^3^The images of the *YorkUrbanDB* dataset have a resolution of 640 × 480. Thus, the real values of *d* and *p* range between −400 and 400.

^4^Higher ratios than those presented in Bachiller-Burgos et al. (2017) can be observed due to two main factors. The first one is that, in this study, we use a rounded integer distance to accept matchings between detected and ground truth endpoints. In addition, ground truth endpoints situated at a distance less than 1 pixel from each other are considered to be situated at the same image position, which reduces the size of the validation set.
